# Picking Towels in Point Clouds

**DOI:** 10.3390/s19030713

**Published:** 2019-02-10

**Authors:** Xiaoman Wang, Xin Jiang, Jie Zhao, Shengfan Wang, Tao Yang, Yunhui Liu

**Affiliations:** 1 Mechanical Engineering and Automation, Harbin Institute of Technology, Shenzhen 518055, China; 16b953039@stu.hit.edu.cn (X.W.); zhaojie@stu.hit.edu.cn (J.Z.); 18S053234@stu.hit.edu.cn (S.W.); yangtao@stu.hit.edu.cn (T.Y.); 2 Harbin Institute of Technology, Harbin 150001, China; 3 Department of Mechanical Engineering, The Chinese University of Hong Kong, Hong Kong, China

**Keywords:** picking towels, convex wrinkles, point clouds, grasp pose

## Abstract

Picking clothing has always been a great challenge in laundry or textile industry automation, especially when some clothes are of the same colors, material and entangled with each other. In order to solve the problem, we present a grasp pose determination method to pick towels placed in a laundry basket or on a table. In our method, it is not needed to segment towels into independent items and the target towels are not necessarily distinguishable in color. The proposed algorithm firstly segments point clouds into several convex wrinkles, and then selects the appropriate grasp point on the candidate convex wrinkle. Moreover, we plan the grasp orientation with respect to the wrinkle which can effectively reduce the grasp failure caused by the inappropriate grasp direction. We evaluate our method on picking white towels and square towels, respectively, and achieved an average success rate of about 80%.

## 1. Introduction

Robots have always played an important role of industrial automation production. However, most industrial robots focus on dealing with rigid objects. So far, there are many methods to solve the problems about picking the rigid objects. However, these methods cannot be directly applied to non-rigid objects. This requires robots to acquire new techniques to automate the operation of soft deformed objects. Because of the flexibility of the deformed objects and the arbitrariness of their appearance, it is difficult to estimate their state. Despite these difficulties, great progress has been made in the field in recent years, which can be referred to in the literature [1]. In our daily life, many objects are deformable, such as towels, clothes, socks, sheets, etc. Thus, the problem tackled in the paper is very useful in the textile industry, laundry industry and home service industry.

Whether in the laundry or home service industry, laundry robots should be able to separate clothes by their color, material and category. In addition, after washing and drying the clothes, the laundry robots also need to fold them. Therefore, the current research mainly focuses on classifying and folding of clothing. Based on the 2.5D feature, a single-shot method [2] is used to identify the category of clothes. In the literature [3], classification of clothes is implemented by perceiving the material characteristics of clothes with tactile sensing. Hanging clothes are classified by using interactive perception [4]. Based on the idea of [5], a convolutional neural network architecture is introduced to realize the classification of the hanging garments [6]. The algorithm [7], L-M-H, is proposed to classify clothes and the research indicates that the middle layer plays an important role in the final classification results. Some research [8,9,10] tackles the problem of folding clothes on a table. In addition, some studies are about the complete folding process—for example, [11].

**Figure 1:**
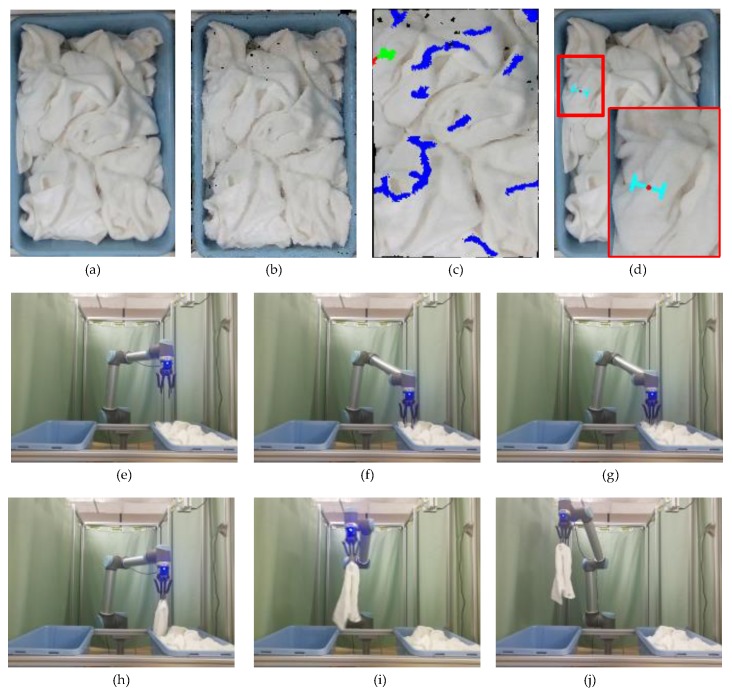
The complete process of the method: (**a**) the RGB image from Realsense D435; (**b**) the point clouds of the white towels; (**c**) the appropriate grasp point on the candidate convex wrinkle; (**d**) the projection of the grasp pose on the image; (**e**) the robotic arm reaches the pre-grasp position; (**f**) the robotic arm reaches the grasp position; (**g**) the robotic arm closes two-finger gripper; (**h**) the robotic arm picks a towel; (**i**) the robotic arm moves the towel from the right laundry basket to the left laundry basket; (**j**) the robotic arm stands by placing.

Although there are many studies on clothing manipulation, as far as I know, there is still a long way to realize its automation. For example, in the laundry industry, after washing the towels and sheets, it is still necessary to manually pick one from a pile of messy towels or sheets, and then feed it to the folding robot [12]. Like the situations in the home service industry, after washing, you can use the robot “laundroid” [13] to automatically fold and organize clothes. In the process of using it, you only need to throw several items of clothes, and the robot “laundroid” [13] will carry out the operations of grasping, spreading, folding, sorting, etc. However, it can fold only one piece of clothing at a time, and the whole process takes about 5 to 10 min. No matter what applications, picking a cloth is the essential step. In the large-scale laundry industry, the main objects of washing are towels and sheets. Thus, in this paper, we mainly study picking towels. In order to provide a more general solution, the colors of the towels are chosen to be white, and they are also made of the same materials, as shown in Figure 1a. Because there are thousands of shapes of the towels without restrictions, their perception is very difficult. In addition, these clothes are highly deformed and entangled, which brings a great challenge to picking.

Determining a grasp point is a crucial issue in the manipulation of clothing. Therefore, many ways have been proposed to search the grasp point. A wrinkle measurement method [14] for textiles is proposed, and the grasp point is the point with higher activation on the map. Willimon et al. [4] firstly estimate the top item of a pile of clothes, and then take the geometric center of the top item as the grasp point. Ramisa et al. [15] cannot only recognize specific clothes wrinkles, but also detect a reliable grasp point. Hata et al. [16] take the top position of a pile of clothes as the grasp point. A Bag of Features [17] based on appearance and 3D geometry is built, and a good grasp point on the collar is found. Bersch et al. [18] take the highest point of the T-shirt on the table as the grasp point. Both the highest point and the most wrinkled point [14] are used as the desired grasp point in [19]. Although it is easier to use the highest point as the grasp point, there is no guarantee that one towel will be grasped successfully, especially when the highest point is on a flat surface of the towels. This paper chooses the point on the convex wrinkle as the grasp point.

In order to pick one from a pile of clothes of the same color and material, it needs to find other ways except the one simply relying on RGB information. Thus, in this paper, we use point clouds to extract the convex wrinkle features, select the appropriate grasp point on the candidate convex, and realize the picking. In our experiment, we find that the towels have both convex and concave wrinkles, and that choosing a grasp point on the convex wrinkle can increase the success rate of picking towels.

In the paper, we realize picking towels based on point clouds, as shown in Figure 1. Our main contributions of the article are as follows: (1) We proposed a grasping planning method for towel-like deformable objects randomly placed. The method utilizes point clouds from the sensor as input. Different from the other research, it chooses convex wrinkles demonstrated in the point cloud as the features for tackling a planning problem. (2) In this research, not only the position of grasp point but also the pose of the gripper are tackled. In the other related research, pose of grasping is not considered since, for highly deformable objects, it is assumed that the objects will deform adaptively to facilitate grasping. In this paper, the authors have proved that, even for highly deformable objects like towels, it is necessary to plan the grasping pose delicately. The proposals above help to increase the robustness and efficiency for grasping randomly placed highly deformable objects. Its effectiveness has been proved in the experiments.

## 2. Methods

In order to find the wrinkles of the object, we treat the point clouds as a “graph”, and then use the Graph-Based algorithm [20] to segment the convex wrinkles. However, it should be noted that this “graph” is not organized. In addition, it turns out that this method can achieve good results. The main process of the method is presented in Algorithm 1.

**Algorithm 1:** Determining Grasp Pose(x,y,z,w)  **Input**: point clouds, C; a region of interest, R 
  **Output**: Grasp Pose(x,y,z,w)   step 1: R=Get_ROI(C);  step 2: G_normal=Get_normal(R);  step 3: Get the convex wrinkles, *W*, and the number of the convex wrinkles is *M*; (*W*,*M*)=Graph_based_point_clouds(G_normal);  step 4: Candidate convex wrinkle, which has the most number of convex points;  Cd=Most_Convex(*W*,*M*);  step 5: Pose(x,y,z,w)=Get_Grasp_Pose(Cd);


In Algorithm 1, step 1 determines the region of interest where we want the robot to pick the towels. It is important to note that we do not need to segment the object from the background, but the method used in Ref. [2] necessitates it. Moreover, in Ref. [2], it requires that the color of background and that of the clothing is different. Step 2 contributes to calculating the normal vector of the point clouds. Step 3 is used to obtain the convex wrinkles based on the Graph-Based algorithm [20]. The method of the paper is partly inspired by the literature [21,22]. In Step 4, the convex point with the highest number of points is considered as the candidate wrinkle. In Step 5, the appropriate grasp point is selected from the candidate wrinkles.

**Figure 2:**
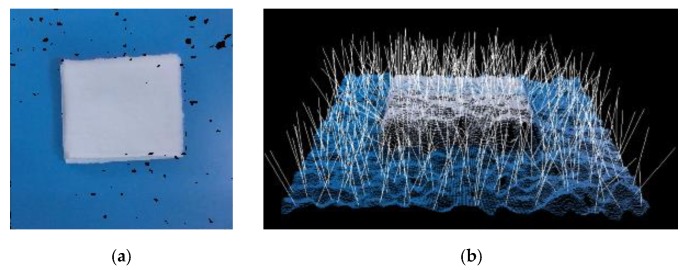
The sign of the normals: (**a**) the point clouds of the towel on a table; (**b**) the point clouds of the towel and its normals.

In this paper, we will introduce the last three steps of Algorithm 1 and with the introduction to steps 1 and 2 skipped. Determining the region of interest is mainly to extract the region containing the towels, and to eliminate the interference caused by the background. In step 2, the normal vector of the point clouds is calculated by the Point Cloud Library (http://pointclouds.org/). After the normals obtained, we need to determine the sign of the normals. From Ref. [23], we know that there is no mathematical method to decide the sign of the normal. Just like in Ref. [23], we define the normals outward from the towel as positive, as shown in Figure 2.

### 2.1. The Concave and Convex Criterion

**Figure 3:**
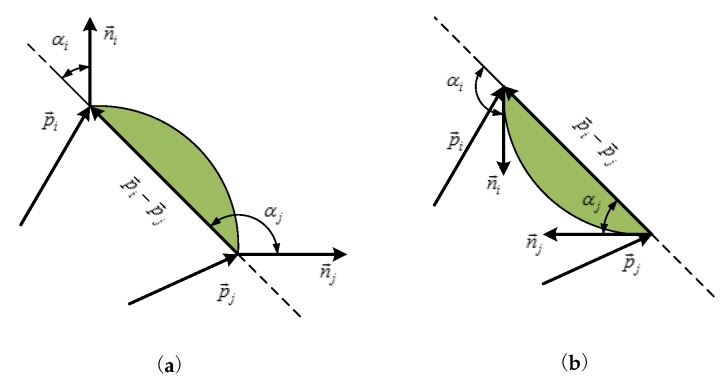
The concave and convex criterion: (**a**) the convex surface; (**b**) the concave surface.

The idea of the concavity and convexity criterion mainly comes from LCCP [24]. It is mainly based on the relationship between the normal vectors $\vec{n_{i}}$, $\vec{n_{j}}$ and the vector ($\vec{p_{i}}-\vec{p_{j}}$) to judge whether the surface between the point $\vec{p_{i}}$ and the point $\vec{p_{j}}$ is convex or concave, as shown in Figure 3. If the angle between the normal $\vec{n_{i}}$ and the vector ($\vec{p_{i}}-\vec{p_{j}}$) is smaller than the angle between the $\vec{n_{j}}$ and the vector ($\vec{p_{i}}-\vec{p_{j}}$), there may be convex surface between the point $\vec{p_{i}}$ and $\vec{p_{j}}$. Conversely, it is a concave surface. As mentioned in LCCP [24], there exist noises from the obtained RGB-D data. It is necessary to add a bias β to ignore some small concave surface, that is, to treat the small concave surface as a convex surface. In our work, we would like to find the more distinct convex surfaces. If the convex surface is not obvious, we regard it as a concave surface, which is the opposite of [24]. In this paper, for the bias $\beta =|\alpha_{i}-\alpha_{j}|$, we choose $\beta_{thresh}=15$.

### 2.2. The Edge Weights

**Figure 4:**
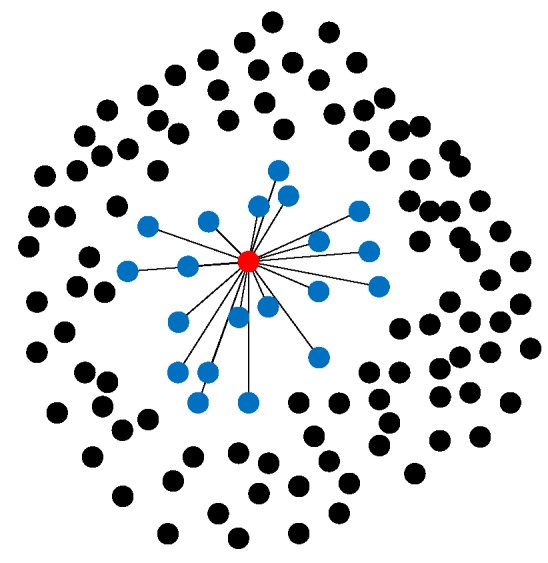
The neighbor points: the red point is the point $\vec{P_{1}}$, the blue points are the neighbor points.

**Figure 5:**
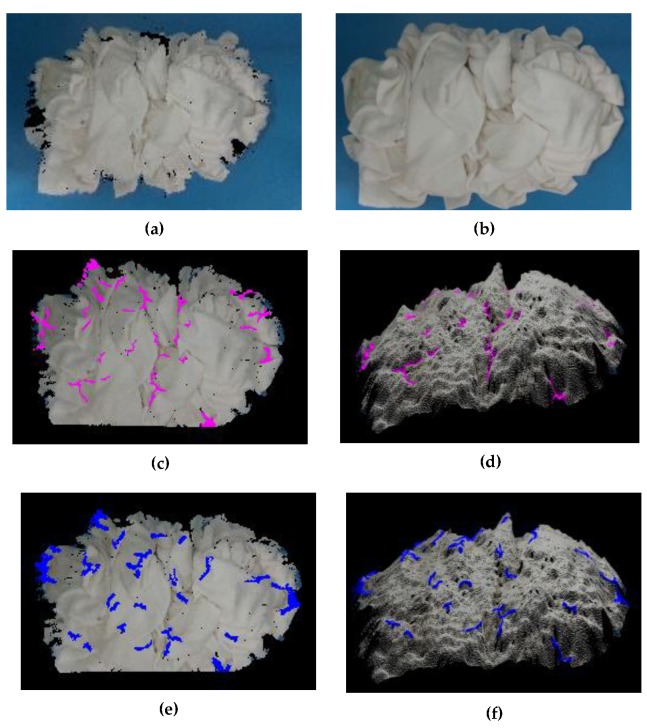
The concave and convex wrinkle: (**a**) the RGB image from Realsense D435; (**b**) the point clouds of the white square towels; (**c**) the concave wrinkle; (**d**) another viewpoint of (**c**); (**e**) the convex wrinkle; (**f**) another viewpoint of (**e**).

The image segmentation algorithm “Graph-Based Image Segmentation” [20] selects the difference in color, intensity or some other local features between two pixels as edge weights. In this paper, we treat the point clouds as a “graph”, but the point clouds are unorganized. The towels are of the same color, so the color information is useless. In addition, instead of segmenting the objects into independent items, we segment the objects into concave or convex wrinkles. Thus, the image-based features we described above cannot be used to calculate weights. In addition, the dot product of the normal vector of the two points is set as the edge weights in this work. This idea mainly comes from [21]. In the image segmentation, we need to calculate the edge weights between a pixel and its neighbor pixels. The neighbor pixels are usually four neighbors or eight neighbors. However, in this work, after the original point clouds are preprocessed, the point clouds become unorganized. Thus, it is impossible to search for four neighbors or eight neighbors of a point. The KNN algorithm (http://pointclouds.org/) is used to select the neighbor points, and the neighbor points of the point $\vec{P_{1}}$ are shown in Figure 4. The local convexity or concavity is most likely a convex or concave wrinkle of the towels. Based on the extended convexity criterion in [24], we set different judgment conditions to obtain concave or convex wrinkles, which are shown in Figure 5. If edge weight is as Equation (1), concave wrinkles can be obtained. In addition, if the condition is as Equation (2), the convex wrinkles can be segmented. In this paper, we choose Equation (2) because the concave wrinkle is not easy for grasping by a two-finger gripper:

(1) $$w_{ij}=\left\{\begin{array}{c} {\vec{n}}_{i}\cdot {\vec{n}}_{j}\;\;\;if\left({\vec{n}}_{i}\right.-{\vec{n}}_{j})\cdot \left({\vec{p}}_{i}-{\vec{p}}_{j}\right.)>0\;\;or\;\beta <\beta_{thresh},\\ -{\vec{n}}_{i}\cdot {\vec{n}}_{j}\;\;\;otherwise, \end{array}\right.$$

(2) $$w_{ij}=\left\{\begin{array}{c} {\vec{n}}_{i}\cdot {\vec{n}}_{j}\;\;\;if\left({\vec{n}}_{i}\right.-{\vec{n}}_{j})\cdot \left({\vec{p}}_{i}-{\vec{p}}_{j}\right.)<0\;\;or\;\beta <\beta_{thresh},\\ -{\vec{n}}_{i}\cdot {\vec{n}}_{j}\;\;\;otherwise. \end{array}\right.$$

### 2.3. Threshold Function T(C)=k/|C|

**Figure 6:**
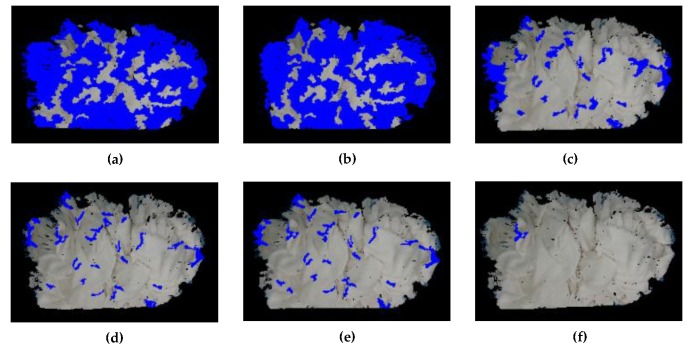
The segmented convex wrinkles with different *k*: (**a**) *k* = 0.5; (**b**) *k* = 0.9; (**c**) *k* = 0.99; (**d**) *k* = 1; (**e**) *k* = 3; (**f**) *k* = 1000.

The Graph-Based algorithm [20] merges two components C1,C2 when the difference between two components is less than the minimum internal difference of the two components. The results of segmentation by the Graph-Based algorithm [20] are related to the *k* of the threshold function. The *k* is a constant parameter that is set according to different situations and the |C| is the size of a component C1 or C2. Figure 6 shows the experiment results when *k* is in different values. If *k* is too small or too large, it will cause the segmentation of the concave and convex wrinkle to fail. The experiment shows that better convex results can be obtained when *k* is between 1 and 10. In this paper, we choose k=3. Following the implementation of “Graph-Based Image Segmentation” [20], we set a hard threshold on the minimum number of points min_size that can merge any smaller wrinkles to neighbor wrinkles. We choose min_size=350. In order to successfully pick the towel, we take the assumption: if the number of the points on the convex wrinkle is less than 400, this convex wrinkle will not be considered as a candidate wrinkle.

## 3. Picking the Towels

### 3.1. Grasp Point P

The grasp point should satisfy that the robotic arm can only grasp one towel at a time. The process of determining the grasp point is presented in Algorithm 2.

**Algorithm 2:** Determining the grasp point P(x,y,z)

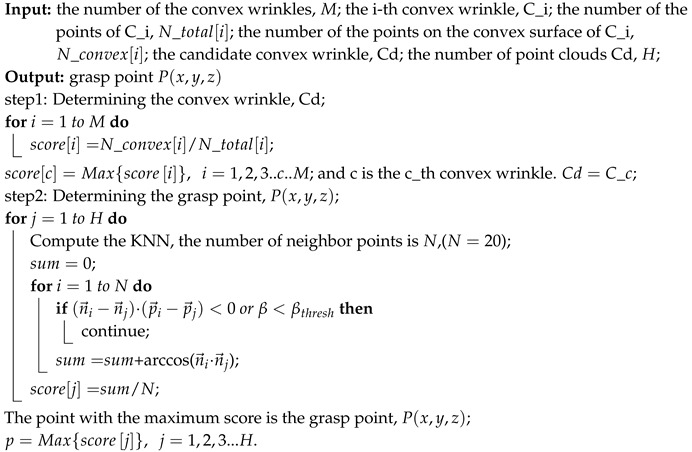



According to step 3 of Algorithm 1, the number of convex wrinkles obtained is presented as *M*. We determine the candidate wrinkle and the process is shown in Algorithm 2. The idea of determining the candidate wrinkle is mainly from [21]. We then select the grasp position P(x,y,z) on the candidate wrinkle. We first traverse all the points on the candidate wrinkle, and find the neighbor point ${\vec{p}}_{i}$. If the surface between the point ${\vec{p}}_{i}$ and ${\vec{p}}_{j}$ satisfies the condition $\left({\vec{n}}_{i}-{\vec{n}}_{j}\right)$·$({\vec{p}}_{i}-{\vec{p}}_{j})<0$ or $\beta <\beta_{thresh}$, we think that there is a concave surface. Otherwise, we think that there is a convex surface. We only compute the angle between the ${\vec{n}}_{i}$ and ${\vec{n}}_{j}$, where there is a convex surface. According to step 2 in Algorithm 2, we obtain a score for each point. In addition, the point which has the maximum score is chosen as the grasp point.

### 3.2. The Grasp Orientation

**Figure 7:**
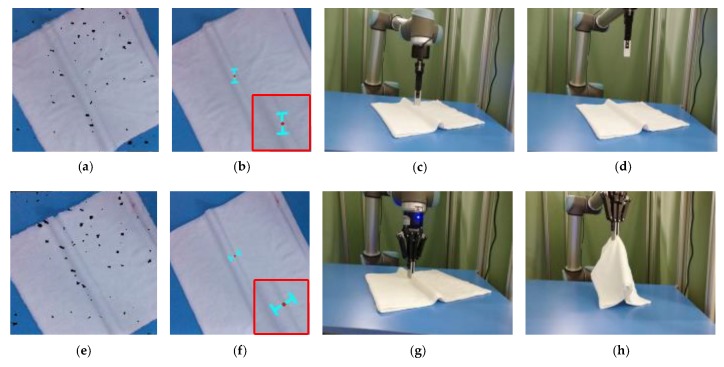
The comparison of good and bad grasp directions: (**a**) the point clouds of a white towel on a table; (**b**) the projection of a inappropriate grasp pose on the image; (**c**) the robotic arm reaches the grasp position; (**d**) the robotic arm picks empty; (**e**) the point clouds of a white towel on a table; (**f**) the projection of a appropriate grasp pose on the image; (**g**) the robotic arm reaches the grasp position; (**h**) the robotic arm picks a towel.

**Figure 8:**
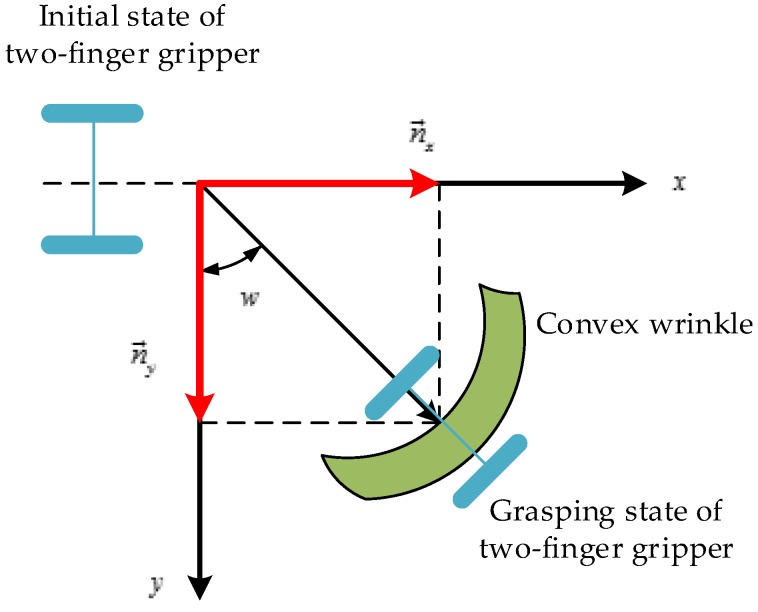
The grasp orientation of the method.

Apart from searching the grasp point, determining the grasp orientation is also important. Although there are many research works studying the grasp pose detection, for example, Refs. [25,26] which are based on the object’s point clouds to detect the grasp pose, their methods are suitable for grasping the rigid objects or soft objects with less deformation. However, the towels are highly deformed, and can be present in multiple shapes. If the grasp orientation is not suitable, the robotic arm is very likely to flatten the wrinkle and result in an air grasping. as shown in Figure 7. Then, the shape of the wrinkle will be changed, and the current grasp pose of the towels will also be changed. This will lead to a grasp failure. Thus, it is important to choose the appropriate grasp orientation. In this paper, in order to successfully pick the towels, we choose the grasp direction along the optical axis of the 3D camera and the opening direction of the two-finger gripper should be perpendicular to the wrinkle, as shown in Figure 7. The rotation angle $w=arctan({\vec{n}}_{x}/{\vec{n}}_{y})$, and the ${\vec{n}}_{x},{\vec{n}}_{y}$ are the normal vectors of the grasp point, as shown in Figure 8. On the other hand, if the grasp path planning is not suitable, it also may lead the gripper to touching the towel. In this paper, we control the arm firstly to reach the pre-grasp position, as shown in Figure 1e, and then reach the grasp-position as shown in Figure 1f, which can avoid the problems above and improve the success rate of the grasp. In addition, it should be noted that the size of the candidate wrinkle is relatively small, so there is a limitation to the distance of the two-finger gripper. If the opening width of the two-finger gripper is too large, it will cause multiple towels to be grasped at a time. In the actual grasp, the appropriate opening width of the two-finger gripper should be selected according to the size of the object. In the experiments, we observe that the width of the wrinkles is generally less than 20 mm, so we determine the opening width of the two-finger gripper as 30 mm.

## 4. Results

**Figure 9:**
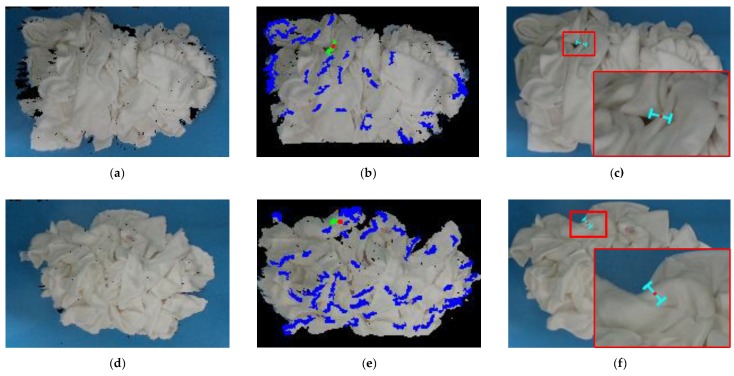
The grasp pose in the scene 1: (**a**) the point clouds of the white towels on a table; (**b**) the grasp point on the candidate convex wrinkle; (**c**) the projection of a grasp pose on the image; (**d**) the point clouds of the white square towels on a table; (**e**) the grasp point on the candidate convex wrinkle; (**f**) the projection of a grasp pose on the image.

**Figure 10:**
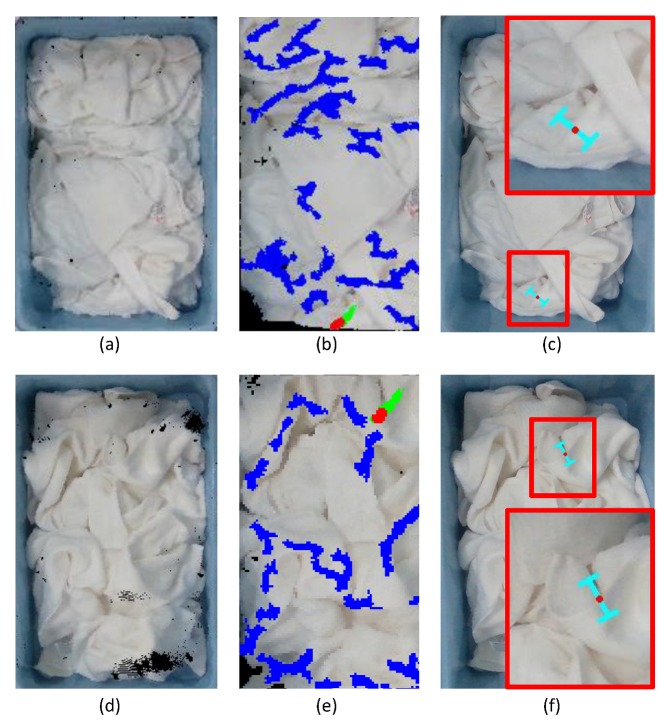
The grasp pose in scene 2: (**a**) the point clouds of the white towels in a laundry basket; (**b**) the grasp point on the candidate convex wrinkle; (**c**) the projection of a grasp pose on the image; (**d**) the point clouds of the white square towels in a laundry basket; (**e**) the grasp point on the candidate convex wrinkle; (**f**) the projection of a grasp pose on the image.

We validated our method in two different scenes: picking the towels placed on a table or placed in a laundry basket. In each scene, we pick 20 white towels and white square towels, respectively. In addition, the white towel and white square towel are different in size and thickness. Ten experiments were performed in each scene. The grasp poses are presented in Figure 9 and Figure 10, respectively, and some grasp results are presented in Table 1, Table 2, Table 3 and Table 4. Figure 9 and Figure 10 show the good grasp pose. In addition, the colors of obvious convex wrinkles are blue. The color of the candidate wrinkle is green. The grasp point is set to red. Since a single point is difficult to see in the point clouds, we set the grasp point as the center of a sphere whose radius is 0.01 m, and the color of the sphere is red for the display. We achieved the grasp success rate of about 80% in picking the towels (692 grasp successes out of 868 grasp attempts).

**Figure 11:**
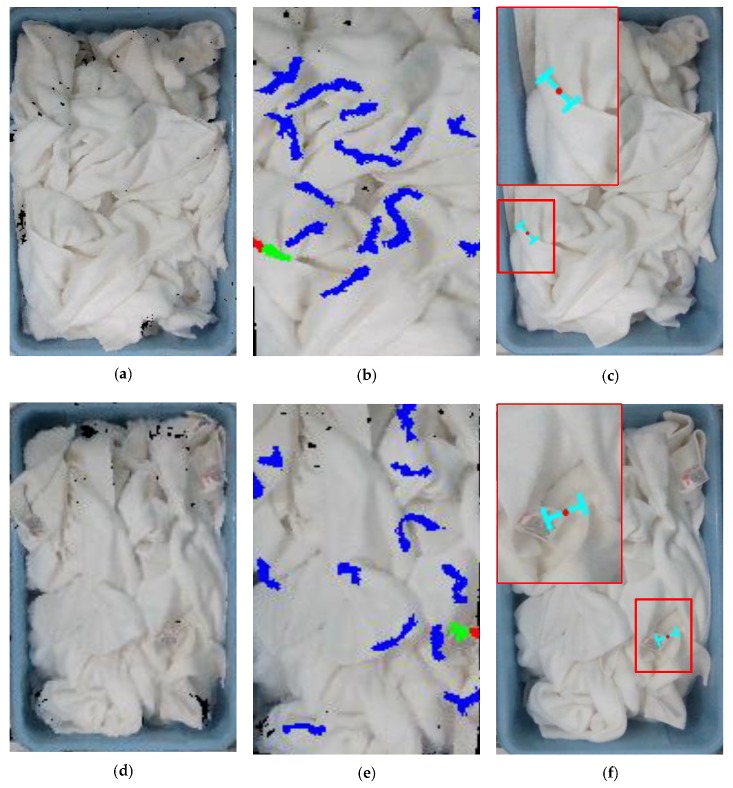
The examples of grasp failure: (**a**) the point clouds of the white towels; (**b**) the grasp point on the candidate convex wrinkle. (**c**) the projection of a grasp pose on the image, and the grasp pose is inappropriate; (**d**) the point clouds of the white towels; (**e**) the grasp point on the candidate convex wrinkle; (**f**) the projection of a grasp pose on the image, and the grasp point is in the trademark.

The experiments confirm that our method enables picking the towels in two different scenes. However, there are picking failures, which we analyze here. The grasp failure is mainly from grasp empty. In addition, the grasp empty is mainly due to the noise in the point clouds and not high enough accuracy of the sensor, which led to the incorrect calculation of grasp orientation, as shown in Figure 11c. Some failures come from the inappropriate grasp position where the grasp point is in the trademark, as shown in Figure 11f. Because the trademark is thin and smooth, the towel falls down after an initially successful grasp. Other failures will appear when the candidate wrinkle is located where two towels overlap.

**Figure 12:**
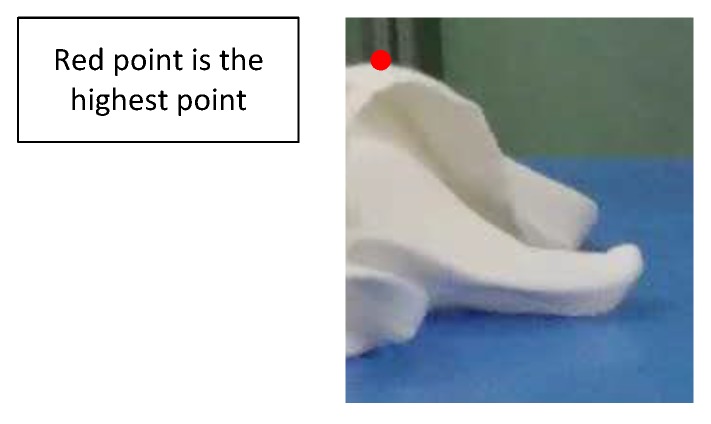
The highest point is on a flat surface below which there is nothing.

**Figure 13:**
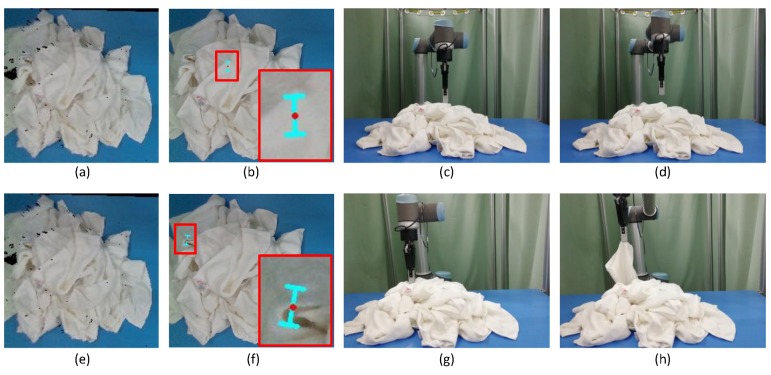
The comparison of the highest point and our method: (**a**) the point clouds of the white square towels on a table; (**b**) the projection of a grasp pose on the image with the highest point; (**c**) the robotic arm reaches the grasp position;(**d**) the robotic arm picks empty; (**e**) the point clouds of the white square towels on a table; (**f**) the projection of the grasp pose on the image with our method; (**g**) the robotic arm reaches the grasp position; (**h**) the robotic arm picks a towel.

As far as I know, selecting the highest point for grasping the clothes is also a common method. In Ref. [16], the highest point in the point cloud is selected as the grasp point for towels, and it is also reported that a success rate of 94.1% is achieved in their experiments. In the literature [16], no detailed information such as how success grasping is defined on the experiments is provided. In addition, the sensors, robot gripper, and towels employed in those experiments are different from those used in our research. In order to make a comparison between their method and our method, we have conducted a set of experiments on our system. The results of the experiments are summarized in Table 5 and Table 6. The situation demonstrated in Figure 12 explains the drawback of choosing the highest point as the grasp point well. In this case, the highest point estimated in the point cloud is on a flat surface. Actually, below the target towel which contains the highest point, there is nothing. Grasping of the point will most likely function to push the towel and result in an air grasping. In the next grasping try, it is still possible that the highest point is selected from the same towel. In our experiments, the maximum number of air grasping can be up to 10 as shown in Figure 13. In the comparison experiments, 20 successive tries are permitted for both of the methods to pick a pile of towels. The robot attempts to pick the towels one by one. A successful grasping is defined as the try successfully grasping only one towel. The ratio of the number of successful tries to that of the total number of tries is then defined as the success rate. In the experiments, the robot gripper will approach the towels along the *z*-axis. The opening direction of the two-finger gripper is shown in Figure 13c. The experiment containing 20 grasping tries are repeated for five different settings of towels.

## 5. Discussion

Based on the point clouds, this paper tries to select the appropriate grasp pose from a pile of towels with the same color. There are many ways to determine the grasp pose of clothing. For example, Sun et al. [2] segment a pile of clothes into independent items, and then find a suitable grasp pose on the selected clothes. Unfortunately, the method of Ref. [2] requires that the clothes are different colors. Instead, we determine the grasp pose in point clouds, and this method does not need to segment a pile of towels into independent items and to know the color information. This leads to an obvious advantage. The reasons are that correctly segmenting clothes into independent items is a difficult problem, and segmentation failure is also likely to cause picking failure. The other superior point of our approach is that we also plan the grasp orientation with respect to the wrinkle, which can effectively reduce the grasp failure caused by the inappropriate grasp direction, as shown in Figure 7. In our experiment, we achieved a grasp success rate of about 80% in picking the towels. In fact, the grasp success rate is an average over a large number of picking experiments (in total, 692 grasp successes out of 868 grasp attempts). However, there are two drawbacks for determining the grasp pose in point clouds. The first one is that our method is sensitive to the k value of threshold function, as shown in Figure 6. If the k value is not appropriate, it will cause the segmentation of the convex wrinkle to fail. The other one is that, although the grasp point is appropriate, the noises in the point clouds and the insufficient accuracy of the sensor lead to the incorrect calculation of grasp orientation, that is, the two-finger gripper is not always perpendicular to the wrinkle, as shown in Figure 11. We hope to solve the above problems and further improve the success rate in our future work. In addition, the opening width of the two-finger gripper is determined before in advance. Actually, it is possible to determine the opening width by measuring the width of the target wrinkle. We will try to integrate the idea in our future work.

## 6. Conclusions

In this paper, we present a method for picking towels randomly placed one by one. The method utilizes point clouds and it chooses convex wrinkles as the features for determining grasp plan. In this research, the proposed grasp planning method determines not only the position of grasp point but also the pose of the gripper. The effectiveness of the proposed method is verified by experiments.

## Figures and Tables

**Table 1:** Picking the white towels on the table (GN: the number of towels grasped).

Experiment No.	GN = 0	GN = 1	GN = 2	GN = 0,1,2	success_rate
1	3	16	2	21	76.2%
2	3	18	1	22	81.8%
3	4	18	1	23	78.2%
4	6	18	1	25	72%
5	2	16	2	20	80%
6	2	18	1	21	85.7%
7	4	18	1	23	78.2%
8	3	18	1	22	81.8%
9	3	20	0	23	86.9%
10	5	20	0	25	80%
**total**	**35**	**180**	**10**	**225**	**80%**

**Table 2:** Picking the white square towels on the table (GN: the number of towels grasped).

Experiment No.	GN = 0	GN = 1	GN = 2	GN = 0,1,2	success_rate
1	4	18	1	23	78.3%
2	5	18	1	24	75%
3	0	14	3	17	82.3%
4	2	18	1	21	85.7%
5	5	16	2	23	**69.5%**
6	5	20	0	25	80%
7	1	20	0	21	**95.2%**
8	5	14	3	22	63.6%
9	6	20	0	26	76.9%
10	2	18	1	21	85.7%
**total**	**35**	**176**	**12**	**223**	**78.9%**

**Table 3:** Picking the white towels in a laundry basket (GN: the number of towels grasped).

Experiment No.	GN = 0	GN = 1	GN = 2	GN = 0,1,2	success_rate
1	2	16	2	20	80%
2	2	20	0	22	**90.9%**
3	4	20	0	18	83.3%
4	3	14	3	20	70%
5	9	20	0	29	**68.9%**
6	3	18	1	22	81.8%
7	0	16	2	18	88.8%
8	4	18	1	23	78.3%
9	1	16	2	19	84.2%
10	1	14	3	18	77.8%
**total**	**29**	**172**	**14**	**215**	**80%**

**Table 4:** Picking the white square towels in a laundry basket (GN: the number of towels grasped).

Experiment No.	GN = 0	GN = 1	GN = 2	GN = 0,1,2	success_rate
1	2	18	1	21	85.7%
2	1	18	1	20	**90%**
3	1	14	3	18	77.8%
4	1	18	1	20	**90%**
5	5	18	1	24	75%
6	1	16	2	19	84.2%
7	5	16	2	23	**69.5%**
8	4	16	2	22	72.7%
9	1	18	1	20	**90%**
10	2	12	4	18	80%
**total**	**23**	**164**	**18**	**205**	**80%**

**Table 5:** Picking the white square towels on the table with the highest point (GN: the number of towels grasped).

Experiment No.	GN = 0	GN = 1	GN = 2	GN = 0,1,2	success_rate
1	12	8	0	20	40%
2	6	13	1	20	65%
3	1	17	2	20	85%
4	3	15	2	20	75%
5	9	11	0	20	55%

**Table 6:** Picking the white square towels on the table with our method (GN: the number of towels grasped).

Experiment No.	GN = 0	GN = 1	GN = 2	GN = 0,1,2	success_rate
1	3	17	0	20	85%
2	5	15	0	20	75%
3	1	17	2	20	85%
4	8	11	1	20	55%
5	4	15	1	20	75%
